# Impaired mechanical, heat, and cold nociception in a murine model of genetic TACE/ADAM17 knockdown

**DOI:** 10.1096/fj.201801901R

**Published:** 2018-12-26

**Authors:** Serena Quarta, Miodrag Mitrić, Theodora Kalpachidou, Norbert Mair, Natalia Schiefermeier-Mach, Manfred Andratsch, Yanmei Qi, Michiel Langeslag, Philipp Malsch, Stefan Rose-John, Michaela Kress

**Affiliations:** * Division of Physiology, Department of Physiology and Medical Physics, Innsbruck Medical University, Innsbruck, Austria; and; † Department of Biochemistry, Christian-Albrechts-University Kiel, Kiel, Germany

**Keywords:** pain, sensory neuron, neuroimmune interactions

## Abstract

TNF-α-converting enzyme, a member of the ADAM (A disintegrin and metalloproteinase) protease family and also known as ADAM17, regulates inflammation and regeneration in health and disease. ADAM17 targets are involved in pain development and hypersensitivity in animal models of inflammatory and neuropathic pain. However, the role of ADAM17 in the pain pathway is largely unknown. Therefore, we used the hypomorphic ADAM17 (ADAM17^ex/ex^) mouse model to investigate the importance of ADAM17 in nociceptive behavior, morphology, and function of primary afferent nociceptors. ADAM17^ex/ex^ mice were hyposensitive to noxious stimulation, showing elevated mechanical thresholds as well as impaired heat and cold sensitivity. Despite these differences, skin thickness and innervation were comparable to controls. Although dorsal root ganglia of ADAM17^ex/ex^ mice exhibited normal morphology of peptidergic and nonpeptidergic neurons, a small but significant reduction in the number of isolectin β-4–positive neurons was observed. Functional electrical properties of unmyelinated nociceptors showed differences in resting membrane potential, afterhyperpolarization, and firing patterns in specific subpopulations of sensory neurons in ADAM17^ex/ex^ mice. However, spinal cord morphology and microglia activity in ADAM17^ex/ex^ mice were not altered. Our data suggest that ADAM17 contributes to the processing of painful stimuli, with a complex mode of action orchestrating the function of neurons along the pain pathway.—Quarta, S., Mitrić, M., Kalpachidou, T., Mair, N., Schiefermeier-Mach, N., Andratsch, M., Qi, Y., Langeslag, M., Malsch, P., Rose-John, S., Kress, M. Impaired mechanical, heat, and cold nociception in a murine model of genetic TACE/ADAM17 knockdown.

The TNF-α-converting enzyme (TACE) is a protease and member of the ADAM (A disintegrin and metalloproteinase) family and is also known as ADAM17. It cleaves and therefore sheds more than 80 different membrane-anchored proteins and factors. Among them are neurotrophins, TNF-α, TNF receptor, fractalkine, IL-6 receptor, endothelial growth factor receptor (EGF-R) ligands, Notch and molecules engaged in macrophage migration, like L-selectin (1). Several cytokines and growth factors are active when retained on the cell surface and restrict their biologic effects to a specific microenvironment. In addition, soluble receptors, upon shedding from the cell of origin, can regulate the activity of their ligands or antagonize their effect on the same cells as decoy receptors lacking signaling activity.

Global depletion of ADAM17 leads to early embryonic death in most mice (2–4). The few surviving mice are characterized by reduced body weight and epithelial abnormalities, such as open eyelids already at fetus state, eye degeneration, stunted whiskers, and disorganized structure of the hair follicles in the skin (3, 5). Knock-in mice with drastically reduced ADAM17 activity are viable (2). The hypomorphic ADAM17 (ADAM17^ex/ex^) mouse model shows similar deficits as ADAM17 knockout mice, and, furthermore, these mice are highly susceptible to intestinal inflammation (2, 6). The ADAM17^ex/ex^ mice are viable, most likely because they express about 5% of wild-type ADAM17 levels in all tissues (2), maintaining, however, a reduced shedding of ADAM17 substrates. They exhibit compromised eye and heart development and skin defects (2, 3) but, however, no overt changes in the brain, body segmentation, and vascular development. These defects were shown to be linked to the impaired EGF-R signaling, caused by failure of shedding of EGF-R ligands, targets of ADAM17 (2). ADAM17^ex/ex^ mice also show a substantially increased susceptibility to inflammation in dextran sulfate sodium colitis, despite having a normal intestine. They exhibit weight loss, severe inflammation, and ulcerations. The increased susceptibility was shown to be a consequence of the failure to phosphorylate the signal transducer and activator of transcription 3 *via* EGF-R signaling (2). Chemokines and anti-inflammatory cytokines, such as IL-10 and IL-11, were also found to be up-regulated in dextran sulfate sodium–treated ADAM17^ex/ex^ animals (2). This mouse line has been a valuable model for the study of the role of ADAM17 in various disease models, such as atherosclerosis (7), kidney fibrosis (8), small vessel disease (9), and colon cancer (6). Loss-of-function mutations of ADAM17 or some of the factors shed by the enzyme, such as ligands of EGF-R, were identified in patients with inflammatory skin and bowel lesions (10, 11).

Several ADAM17 targets are important regulators of nociception: TNF-α contributes to the development of inflammatory heat hyperalgesia through the sensitization of the ion channel transient receptor potential cation channel, subfamily V, member 1 (TRPV1) (12). Both the ligand binding IL-6 receptor subunit and the IL-6 signal transducer glycoprotein 130 (gp130) are critically involved in the induction of pain and hypersensitivity associated with inflammation, neuropathy, or cancer in mice and rats (13–17). Finally, inhibition of Notch cleavage decreases mechanical hyperalgesia in mice (18).

The strong association of ADAM17 targets with nociception and pain disorders points toward a critical role for the enzyme in the processing of painful stimuli. Based on our previous findings and recent literature, we hypothesized that ADAM17 could be involved in the regulation of nociceptor sensitivity to painful stimuli and of microglia activity. We therefore set out to explore the functional consequences of ADAM17 depletion on signatures of pain sensitivity.

## MATERIALS AND METHODS

### Ethics statement

Experiments were performed according to European Union Directive 2010/63/EU for animal experiments, National Austrian legal requirements, and the recommendations of the International Association for the Study of Pain to minimize animal suffering (19) and with permission of the Austrian Bundesministerium für Wissenschaft, Forschung und Wirtschaft (BMWF-66.011/0102-WF/V/3b/2015).

### Mouse strains

Male wild-type (ADAM17^+/+^) and ADAM17^ex/ex^ mice (between 8 and 13 wk of age) were used in all experiments (2). Briefly, the mice were generated by inserting loxP sites flanking the exon 11, which encodes the catalytic zinc-binding domain. Hypomorphic expression of ADAM17 was achieved by inserting a loxP site together with a cryptic noncanonical donor splice site, downstream of the cryptic acceptor splice site into intron 11, and thereby generating an additional, artificial, exon 11a. Alternative splicing of the artificial exon 11a led to premature disruption of ADAM17 protein translation, as a result of the in-frame stop codon TAG in exon 11a. Tissue-specific Cre recombinase–mediated deletion of exon 11 could be obtained, if needed. In ADAM17^ex/ex^ mice, the level of *ADAM17* mRNA expression is barely detectable because of an insertion of a new exon within the *ADAM17* gene, which induces a translational stop in 95% of the cells (2). Animals were housed on a 12-h light/dark cycle and had free access to food and water.

### Behavioral testing

Animals were maintained in individual cages for the entire duration of the experimental paradigm. Mechanical or heat sensitivity was quantified by standard procedures. All efforts were made to perform experiments in an unbiased and blinded manner; however, strain differences were obvious because of the hypomorphic phenotype of the ADAM17^ex/ex^ mice. In particular, observers were blinded for each set of experiments, and littermate mice were used as controls. Mice were allowed to habituate for 1 h before testing. The lateral side of the plantar surface of the hind paw was stimulated by calibrated von Frey monofilaments with ascending bending forces (1.4, 4, 8, 16, 22.6, 32 and 45.3 mN). The frequency of the paw withdrawal to 5 stimuli for each monofilament was recorded (20). Thermal nociception was assessed using the Hargreaves method (21) and the hot- and cold-plate assays. Briefly, the withdrawal response latency to an increasing heat stimulus (infrared intensity = 51) was obtained with an automated algesiometer (Ugo Basile, Gemonio, Italy) for the Hargreaves test. Before the hot or cold plate assays, mice were habituated on a 30°C heated plate for 5 min. Subsequently, the mice were placed on a plate either heated to 50°C or cooled to 0°C. The time of the first jump and the number of jumps within 3 min were evaluated. Mice were tested on separate days for the different noxious stimuli.

### RNA isolation and quantitative RT-PCR

Dorsal root ganglia (DRG) and spinal cord from adult mice between 8 and 13 wk were isolated and snap-frozen in liquid nitrogen for quantitative RT-PCR. RNA isolation was performed using peqGold TriFast reagent (VWR, Erlangen, Germany) according to the manufacturer’s instruction, followed by a quality check using Nanodrop 2000 (Thermo Fisher Scientific, Waltham, MA, USA). Reverse transcription for mRNA was performed as previously described (20). *ADAM17* gene expression was assessed by quantitative PCR using TaqMan Gene Expression Assay (Mm00456428_m1; Thermo Fisher Scientific). As reference controls, assays for the following genes were used: *hypoxanthine guanine phosphoribosyl transferase* (Mm00446968_m1), *succinate dehydrogenase complex, subunit A, flavoprotein* (Mm01352363_m1), and *transferrin receptor* (Mm00441941_m1; all from Thermo Fisher Scientific). The reactions were prepared according to the manufacturer’s instructions, loaded on MicroAmp Fast Optical 96-well reaction plates (Thermo Fisher Scientific), and placed in the 7500 Fast RT-PCR system (Thermo Fisher Scientific). The PCR cycle protocol used was 10 min at 95°C, 40 2-step cycles of 15 s at 95°C and 1 min at 60°C. Duplicates were run for each sample alongside nontemplate controls. Threshold was set manually at 0.1, whereas baselines were calculated automatically by the software. *ADAM17* expression was provided in relation to the expression of the 3 reference genes using the 2^−^*^ΔCt^*. The mRNA of all 3 reference controls was stably expressed in all samples, as indicated by geNorm (*https://genorm.cmgg.be/*), Normfinder (*https://moma.dk/normfinder-software*), and Bestkeeper (*https://www.gene-quantification.de/bestkeeper.html*) software. No signal was detected in the reverse transcription non-template controls.

### Immunohistochemistry

DRG, spinal cord, and glabrous hind paw skin were dissected and postfixed in Zamboni’s fixative (2% paraformaldehyde and 1.5% picric acid in PBS) for 1 and 16 h, respectively. Tissue was cryoprotected in 25% sucrose in PBS at 4°C for at least 24 h, embedded, and frozen in optical cutting medium (Tissue-Tek OCT; Sakura Finetek, Tokyo, Japan). Cryostat sections (Leica CM 1950; Leica Biosystems, Wetzlar, Germany) of 12 µm for DRG and spinal cord and 20 µm for the hind paw skin were mounted on polylysine coated slides (Thermo Fisher Scientific).

#### DRG and spinal cord

Blocking solution (10% normal goat serum in PBS with 0.3% Triton X-100) and specific primary antibodies were used to visualize the expression of the protein of interest, ADAM17 (1:300, ab39162; Abcam, Cambridge, United Kingdom); to visualize the DRG subpopulations, isolectin β-4 (IB4; 1:500; Thermo Fisher Scientific), calcitonin-related peptide (CGRP, 1:100; Santa Cruz Biotechnology, Dallas, TX, USA), and neurofilament 200 (NF200; MilliporeSigma, Burlington, MA, USA); and to visualize and count microglia, phosphorylated p38 (Thr180/Tyr182, 1:300; Cell Signaling Technology, Danvers, MA, USA) and cd11b (1:100; Bio-Rad, Hercules, CA, USA). Secondary antibodies used were goat α-rabbit Alexa Fluor 594 and donkey α-mouse Alexa Fluor 488 (1:800; Thermo Fisher Scientific) for fluorescence microcopy. Sections were incubated with only secondary antibodies as control for specificity. ADAM17 antibody specificity was confirmed by the manufacturer as reported in the data sheet and by published data from our collaborators (22). Microglia were counted as activated when positive for both cd11b and phosphorylated p38 immunoreactivity and quantified in a defined area of the spinal dorsal horn corresponding to lamina I and II. In particular, the area of the spinal dorsal horn was delimitated in equal manner using the Freehand Selection tool of the ImageJ software (National Institutes of Health, Bethesda, MD, USA; *http://imagej.nih.gov/ij*) in all the acquired pictures, and microglia, double-stained for cd11b and phosphorylated p38, were identified and manually marked using the Point tool. Measurements were performed by a blinded operator.

#### Glabrous skin

Sections were incubated with blocking solution for 1 h, followed by incubation with primary antibodies (anti-Neuron-specific class III beta-tubulin; α-NF200) at room temperature for 12 h. After washing, sections were incubated at room temperature with the appropriate secondary antibodies for 90 min, washed, and embedded in Mowiol (MilliporeSigma) (17). Indirect immune fluorescence was visualized on a Leica SP5 confocal microscope at ×63 magnification with a 1.4 numerical aperture glycerol-immersion objective. Analysis of immunostaining was performed blinded to genotype. Images were processed using ImageJ (v.1.45h) with global adjustments in brightness and contrast. Quantification of epidermal sensory innervation density was performed as previously described (23). In brief, labeled nerve fibers in the epidermis of at least 10 randomly chosen confocal micrographs (20-µm stacks) of 4 animals per genotype were counted, and the fiber density (number of fibers/1000 µm^2^) was calculated. The thickness of the dermis was measured after identifying the different skin layers through the nuclei staining with DAPI (100 ng/ml).

### Western blot

Spinal cord (L3–L5) was harvested from ADAM17^+/+^ and ADAM17^ex/ex^ mice and processed in freshly prepared ice-cold lysis buffer (50 mM Tris-HCl, 200 mM NaCl, 50 mM NaF, 1 mM EDTA, 20 mM β-glycerophosphate, 1% Triton X-100) enriched with 1:30 phosphatase inhibitor complex (MilliporeSigma) and 1:100 phosphatase inhibitor sodium-*ortho*-vanadate (MilliporeSigma). Lysates were briefly sonicated, and protein content was measured with the BCA Protein Assay Kit (Thermo Fisher Scientific), according to the manufacturer’s protocol. SDS-PAGE was conducted under standard denaturing conditions, and 50 µg of protein were loaded per lane. For protein detection, membranes were incubated with the specific antibodies diluted according to the manufacturers’ instructions. Blots were visualized with ECL using the SuperSignal West Pico Chemiluminescent Substrate (Thermo Fisher Scientific), and membranes were scanned with LAS4000 luminescent imager (GE Healthcare, Chicago, IL, USA). Quantification was performed using ImageJ software. Relative values for phosphorylated proteins were related to the nonphosphorylated form. α-phosphorylated-p38 (1:1000; Cell Signaling Technology), α-p38 (1:1000; Cell Signaling Technology), and α-tubulin (1:2000; MilliporeSigma) were used as primary antibodies, and horseradish peroxide–conjugated α-rabbit IgG (1:5000; Thermo Fisher Scientific) and α-mouse IgG (1:10,000; MilliporeSigma) were used as secondary antibodies.

### Skin-nerve preparation and single-fiber recordings

A modified *in vitro* skin-nerve preparation was used to investigate the properties of the afferent nerve fibers innervating the skin of the mouse dorsal hind paw (24–28). In brief, the sural nerve and innervated skin of the hind paw were dissected, and the preparation was placed corium-side–up in an organ bath chamber and superfused (∼12 ml/min) with an oxygen-saturated modified synthetic interstitial fluid solution containing (in millimolars) 108 NaCl, 3.48 KCl, 3.5 MgSO_4_, 26 NaHCO_3_, 1.7 NaH_2_PO_4_, 2.0 CaCl_2_, 9.6 sodium gluconate, 5.5 glucose, and 7.6 sucrose at a temperature of 31.5 ± 0.8°C and a pH of 7.4 ± 0.05. The distal end of the sural nerve was pulled into a separate chamber and electrically isolated from the bath solution using paraffin oil. Fine nerve strands dissected from the nerve bundle were placed on a gold wire recording electrode. Action potentials (APs) were recorded extracellularly, amplified (up to 5000-fold), filtered (low pass 1 kHz, high pass 100 Hz), and visualized on an oscilloscope and stored/analyzed on a PC-type computer with the Spike/Spidi software package (29). The receptive field was first identified by mechanical probing of the skin with a glass rod and later electrically stimulated with square-wave pulses (duration 1 ms; interstimulus interval 5 s) of up to 100 V. Activation threshold and conduction velocity of the nerve fibers were computed. The fibers were characterized as unmyelinated (C-fibers) according to their conduction velocity (<1.0 m/s). The mechanical threshold of each unit was determined with calibrated von Frey filaments with a uniform tip diameter of 0.8 mm by applying increasing forces from 1 mN to 256 mN, starting with a filament of 22.6 mN. The standard heat stimulus linearly increased the intracutaneous temperature from 31 to 50°C within 20 s. The standard cold stimulus decreased the temperature from 31 to 3°C within 4 s and held this temperature for 20 s. Fibers were considered sensitive if 5 or more APs were evoked during the stimulus. The threshold was defined as the force or temperature that elicited the second spike of the response. Spontaneous activity of isolated C-fibers was determined over 1 min before mechanical and heat stimuli were applied. C-fibers showing 4 or more APs over 1 min were considered spontaneously active.

### DRG primary culture

Lumbar DRG were harvested from adult ADAM17^ex/ex^ and ADAM17^+/+^ mice and prepared as previously described (14, 30). Briefly, ganglia were incubated in Liberase Blendzyme 1 (9 mg/100 ml DMEM; Roche, Basel, Switzerland) for 2 times 30 min, washed, and incubated with Trypsin-EDTA (Thermo Fisher Scientific) for 15 min. After washing with supplemented TNB medium (Biochrom, Berlin, Germany) with l-glutamine (Thermo Fisher Scientific), penicillin G sodium, streptomycin sulfate (Thermo Fisher Scientific), and Protein-Lipid-Komplex (Biochrom AG), DRG were dissociated and centrifuged through a 3.5% bovine serum albumin gradient. The sensory neurons were resuspended and plated on coverslips coated with a mixture of poly-L-Lysine/laminin (MilliporeSigma) and kept in TNB medium containing protein lipid complex, supplemented with 25 ng/ml nerve growth factor (NGF) (Alomone Labs, Jerusalem, Israel) at 37°C in a humidified incubator with 5% CO_2_.

### Patch-clamp recordings

Neurons were used for electrophysiology 18–24 h after seeding. Neurons were visualized using a Zeiss Axiovert 200 microscope (Carl Zeiss, Jena, Germany), and small to medium size diameter (<35 μm) neurons with no apparent neurites were selected. Patch pipettes were pulled from borosilicate glass capillary (Science Products, Hofheim am Taunus, Germany) using a flaming micropipette puller (p97; Sutter Instrument, Novato, CA, USA). Patch pipettes had a tip resistance of 3–6 MΩ. The internal pipette solution contained (in millimolars) 98 K-gluconate, 45 KCl, 2 MgCl_2_, 0.5 CaCl_2_, 10 HEPES, 5 EGTA, 2 MgATP, and 0.2 NaGTP (pH adjusted to 7.3 with KOH). Neurons were kept in an extracellular solution containing (in millimolars) 145 NaCl, 5 KCl, 2 CaCl_2_, 1 MgCl_2_, 10 HEPES, and 10 D-glucose (pH adjusted to 7.3 with NaOH). All measurements were performed at room temperature with an EPC 10 USB Double (Heka, Lambrecht, Germany) and the PatchMaster software v.2x73.1 (Heka).

Voltage recordings were performed in the whole-cell current-clamp mode of the patch clamp technique. In order to assess the passive membrane properties, the input resistance of recorded DRG neurons was determined by a series of −5 pA hyperpolarizing current injections from a holding current of 0 pA (sampled at 5 kHz). The average resistance was calculated according to Ohm’s law. A series of 5 pA, 50 ms depolarizing current injects from a 0 pA holding current was applied to determine the minimal current injection necessary to evoke a single AP (*I*_AP_) in DRG neurons (sampled at 40 kHz). Only neurons that exhibited a hump in the repolarization phase of the AP as a typical feature of a DRG nociceptor AP were analyzed. AP parameters, *e.g.*, resting membrane potential (RMP), afterhyperpolarization (AHP), threshold, AP speed of depolarization and repolarization, and the corresponding time points were analyzed as previously described (31, 32). AHP recovery time constant was derived from fitting the membrane potential recovery after AHP with the single exponential function of an AP evoked by 2 ms 500 pA depolarizing current injection (sampled at 10 kHz). To assess neuronal excitability, series of 5 s ramp-shaped current injections (final current amplitude: 1×, 2×, and 3× *I*_AP_) and a 20 s square-shaped (2× *I*_AP_) current injection were applied (sampled at 20 kHz), and the number of generated APs was determined.

Mechanical stimulation was performed as previously described (33, 34). Briefly, mechanical stimuli were applied using a heat-polished glass pipette (tip diameter 3–5 µm), driven by a MM3A Micromanipulator system (Kleindiek Nanotechnik, Reutlingen, Germany), and positioned at an angle of 45° to the surface of the dish. For soma indentation, the probe was typically positioned so that a 1.4 mm movement did not visibly contact the cell but that a 2.8 mm stimulus produced an observable membrane deflection under the microscope. Therefore, a 2.8 mm probe movement was defined as a 1.4 mm mechanical stimulation, and a series of mechanical steps in 0.7 mm increments were applied at 5 s intervals.

### Microfluorimetric calcium measurements

Calcium imaging was performed as previously described (35). Briefly, cultured cells were nondisruptively loaded with 3 µM of the Ca^2+^-sensitive dye Fura-2 AM (Thermo Fisher Scientific) in TNB medium (Biochrom AG) for 30 min at 37°C. Then cells were washed and kept in Na^+^-free extracellular solution containing (in millimolars) 145 N-methyl-D-glucamine, 5 KCl, 2 CaCl_2_, 1 MgCl_2_, 10 d-glucose (all from MilliporeSigma) and 10 HEPES (MilliporeSigma), at pH 7.3 adjusted with HCl (MilliporeSigma). Experiments were performed using an Olympus IX71 microscope (Olympus, Tokyo, Japan) with a ×20/0.85 numerical aperture oil-immersion objective (Olympus). Fura-2 was excited at 340 and 380 nm (excitation time: 25 ms) with a polychrome IV monochromator (Thermo Fisher Scientific), and fluorescence intensities were filtered by a 510 nm LP filter and recorded with a charge-coupled device camera (CoolSnap; Photometrics, Tucson, AZ, USA). The ratio of fluorescence intensities at excitation wavelengths of 340 and 380 nm (*F*_340/380_) was calculated after background correction, and the changes of intracellular Ca^2+^ signal were depicted as ratio change (Δ*F*_340/380_) measured as peak *F*_340/380_ ratio over baseline. For data acquisition, MetaFluor4.6r8 (Molecular Devices, San Jose, CA, USA) was used, and offline analysis was performed with OriginPro v.7.SR2 (OriginLab, Northampton, MA, USA). All chemicals were diluted in Na^+^-free extracellular solution and applied by a gravity-driven perfusion system (WAS02; Dittel Messtechnik, Landsberg am Lech, Germany). The threshold for a positive response to menthol, cinnamaldehyde (CA) or capsaicin was set to 20% of increase of fluorescent ratio from baseline. Viability of the neurons was tested with a 10 s pulse of 40 mM potassium chloride solution in each experiment.

### Statistical analysis

For statistical analysis the GraphPad (La Jolla, CA, USA) software package v.6 or v.7 was used, and data are presented as means ± sem. For *in vivo* experiments, 2-way repeated measurements ANOVA or Kruskall-Wallis, followed by Tukey *post hoc* test or Mann-Whitney *U* *post hoc* test was used for multiple comparisons between groups for behavioral assays. For *in vitro* data an unpaired Student’s *t* test (Shapiro-Wilk) or Mann-Whitney *U* test with the appropriate correction was performed. Data were analyzed by χ^2^ test for comparison of relative group sizes. Analysis of the AP firing patterns was performed by a 2-way ANOVA with *post hoc* Sidak test for multiple comparisons. Individual tests are reported under each figure legend. Differences were considered statistically significant at *P* < 0.05.

## RESULTS

### ADAM17 localizes to DRG neurons and depletion of ADAM17 affects the IB4-positive nociceptive subpopulation

**Figure 1:**
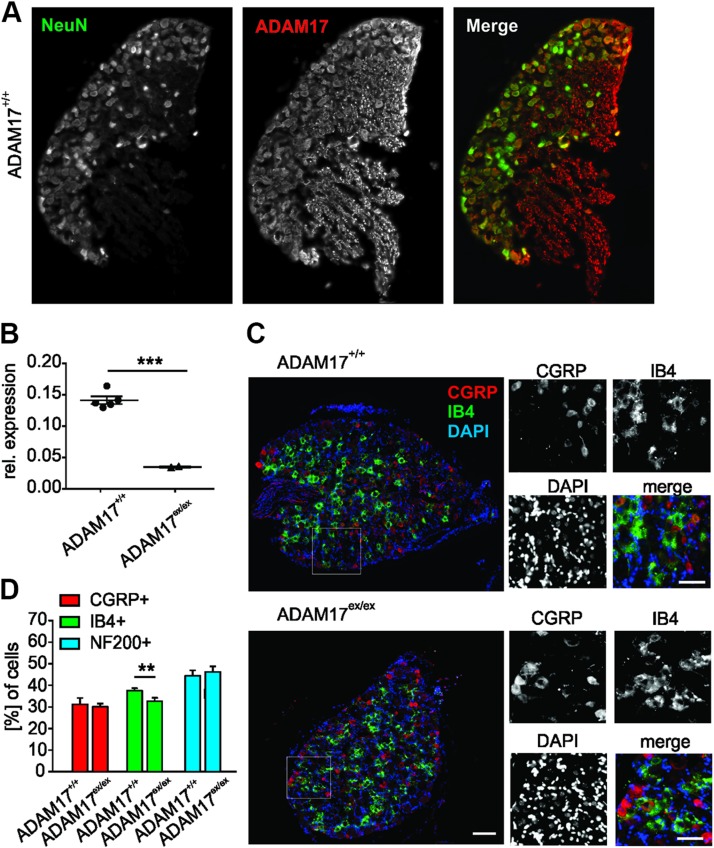
ADAM17 localizes in neurons in DRG, and its down-regulation affects the IB4-positive nociceptive subpopulation. *A*) Expression of ADAM17 in neurons in DRG cryo-sections. Neurons were identified by neuronal nuclei protein (NeuN) staining. Scale bar, 100 µm. *B*) *ADAM17* mRNA was detected in DRG from ADAM17^+/+^ mice and barely in hypomorphic ADAM17^ex/ex^ mice (*n* = 5; unpaired Student’s *t* test with Welch’s correction, *P* < 0.001). *C*) Representative image of DRG stained for the peptidergic [IB4-positive (IB4^+^)] and nonpeptidergic [CGRP-positive (CGRP^+^)] neuronal populations showing similar morphology of ADAM17^+/+^ and ADAM17^ex/ex^ DRG. *D*) The percentage of the nociceptive IB4-positive neurons was found to be significantly decreased in ADAM17^ex/ex^ DRG compared with ADAM17^+/+^ (ADAM17^+/+^ *n* = 3485, ADAM17^ex/ex^ *n* = 2602; χ^2^ test, *P* = 0.0046). The number of CGRP^+^ and NF200-positive (NF200^+^) neurons was similar in the 2 genotypes (CGRP; ADAM17^+/+^ *n* = 2050, ADAM17^ex/ex^ *n* = 3216; χ^2^ test, *P* = 0.5161; NF200; ADAM17^+/+^ *n* = 2050, ADAM17^ex/ex^ *n* = 1756; χ^2^ test, *P* = 0.0775). Scale bars, 200 µm. Results are shown as means ± sem. **P < 0.01, ***P < 0.001.

The ADAM proteins are expressed in different tissues (36); however, 17 mammalian ADAMs are present in the CNS, and many of these are expressed by neurons in largely overlapping regions (37). We found that ADAM17 was predominantly expressed by neurons rather than glia cells in DRG from wild-type (ADAM17^+/+^) mice, and intense immunoreactivity was detected in nerve fibers in DRG sections (**Fig. 1*A***). *ADAM17* mRNA was expressed in DRG neurons from ADAM17^+/+^ mice and was strongly reduced in hypomorphic ADAM17^ex/ex^ mice (Fig. 1*B*). Overall DRG morphology was similar in ADAM17^ex/ex^ and wild-type mice. Despite the comparable morphology of ADAM17^ex/ex^ and ADAM17^+/+^ DRG (Fig. 1*C*), the percentage of nociceptive nonpeptidergic IB4-positive neurons was significantly decreased in ADAM17^ex/ex^ DRG compared with ADAM17^+/+^ (32.55 ± 1.67% for ADAM17^ex/ex^ *vs.* 37.47 ± 1.2% for ADAM17^+/+^; Fig. 1*D*). The percentages of the nociceptive peptidergic CGRP-positive and the non-nociceptive NF200-positive neurons were similar in both genotypes (CGRP-positive neurons: 30.01 ± 1.58% for ADAM17^ex/ex^ *vs.* 31.14 ± 2.98% for ADAM17^+/+^; NF200-positive neurons: 46.16 ± 2.56% for ADAM17^ex/ex^ *vs.* 44.34 ± 2.65% for ADAM17^+/+^; Fig. 1*D*). These results suggest that ADAM17 expression may affect nociceptive neuron subpopulation development and possibly also function.

### ADAM17 down-regulation does not affect spinal dorsal horn architecture or microglia activity

**Figure 2:**
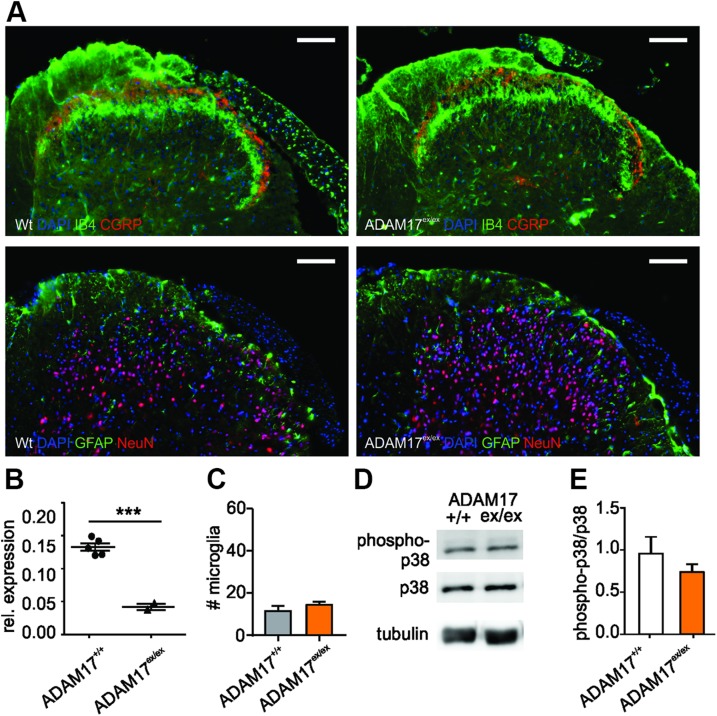
ADAM17 down-regulation does not affect spinal dorsal horn architecture or microglia activity. *A*) Representative staining of the dorsal horn of the spinal cord from ADAM17^+/+^ and ADAM17^ex/ex^ mice. The expression and morphology of nociceptive IB4-positive and CGRP-positive neurons was similar to control animals, as was the distribution of neurons (NeuN-positive) and astrocytes [glial fibrillary acidic protein (GFAP)-positive]. Scale bars, 100 µm. *B*) *ADAM17* mRNA expression was down-regulated in spinal cord from ADAM17^ex/ex^ mice (*n* = 5; unpaired Student’s *t* test with Welch’s correction, *P* < 0.001). *C*) The number of activated microglia was unchanged in the dorsal horn of the spinal cord of ADAM17^ex/ex^ mice in comparison with controls (14.44 ± 1.56 *vs.* 11.44 ± 2.58; *n* = 3; unpaired Student’s *t* test, *P* = 0.3762). *D*) Representative Western blot for the phosphorylated levels of p38 in the dorsal horn of the spinal cord of ADAM17^ex/ex^ and control mice. *E*) Protein levels of phosphorylated p38 (phospho-p38) did not differ between genotypes (0.96 ± 0.2 for ADAM17^+/+^ *vs.* 0.74 ± 0.09 for ADAM17^ex/ex^; *n* = 3; unpaired Student’s *t* test, *P* = 0.3777). Results are shown as means ± sem. ***P < 0.001.

Similar to DRG, the overall architecture of the spinal dorsal horn and in particular the superficial laminae harboring nociceptive IB4- and CGRP-positive fibers as well as the distribution of neurons and glial fibrillary acidic protein–positive astrocytes was similar in ADAM17^ex/ex^ mice and control animals (**Fig. 2*A***), although the applied genetic strategy effectively down-regulated *ADAM17* mRNA expression, not only in DRG but also in the spinal cord (Fig. 2*B*). Moreover, the number of activated microglia in the spinal dorsal horn ADAM17^ex/ex^ mice was also comparable to control animals (Fig. 2*C*; 14.44 ± 1.56 for ADAM17^ex/ex^ *vs.* 11.44 ± 2.58 for ADAM17^+/+^; *n* = 3; unpaired Student’s *t* test, *P* = 0.3762). These results were in line with unaltered levels of phosphorylated p38 MAPK in the spinal dorsal horn of ADAM17^ex/ex^ mice (Fig. 2*D, E*). Our results suggest that the down-regulation of ADAM17 does not introduce defective primary afferent neuron projections into the spinal dorsal horn or affect microglia activity.

### Reduced sensitivity to noxious mechanical stimulation of ADAM17^ex/ex^ mice

**Figure 3:**
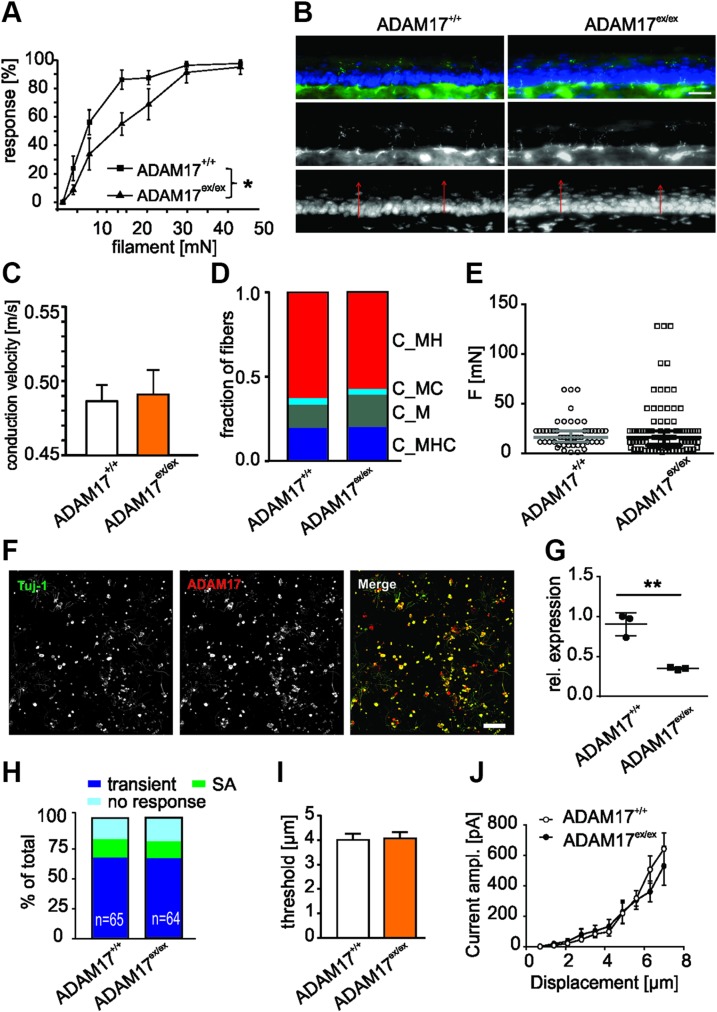
ADAM17^ex/ex^ mice show reduced sensitivity to noxious mechanical stimulation *in vivo*. *A*) ADAM17^ex/ex^ mice showed higher mechanical von Frey thresholds compared with ADAM17^+/+^ animals (*n* = 8; 1-way repeated measurements ANOVA, *P* = 0.0156; Tukey’s *post hoc* test ADAM17^+/+^ *vs.* (ADAM17^+/ex^) *P* = 0.0477; ADAM17^+/+^ *vs.* heterozygous for ADAM17 mice (ADAM17^+/ex^) *P* = 0.2360; ADAM17^+/ex^ *vs.* ADAM17^ex/ex^ *P* = 0.0809). *B*) Representative image of skin sensory innervation. ADAM17^ex/ex^ mice had similar skin innervation pattern (3.08 ± 0.41 *vs.* 2.59 ± 0.16 nerve endings per 1000 µm^2^; *n* = 4; *P* = 0.306, Student’s *t* test) and epidermis thickness (35.10 ± 3.16 *vs.* 37.12 ± 2.54 µm; *n* = 4; *P* = 0.637, Student’s *t* test). Scale bar, 20 µm. *C*) Overall conduction velocities of the 2 genotypes were similar (ADAM17^+/+^: *n* = 38, median 0.49 m/s; ADAM17^ex/ex^: *n* = 54, median 0.46 m/s, *P* = 0.4353, Mann-Whitney *U* test). *D*) C-fibers (C_) are classified by the modalities heat (H), cold (C), and mechanical force (M). The frequency distribution of different C-fiber types shows no statistical significance between the 2 genotypes (ADAM17^+/+^ *n* = 51; ADAM17^ex/ex^ *n* = 88; *P* = 0.8465; χ^2^ = 0.8122, χ^2^ test). *E*) Dot-plot depicting the mechanical threshold of mechanically sensitive C-fibers between ADAM17^+/+^ and ADAM17^ex/ex^ mice (ADAM17^+/+^: *n* = 51, median 16.00 mN; ADAM17^ex/ex^: *n* = 88, median 13.7 mN, *P* = 0.3814, Mann-Whitney *U* test). *F*) Twenty-four hour DRG cultures showed input resistance–positive neurons for ADAM17. Adult neurons are costained for Neuron-specific class III beta-tubulin and ADAM17. Scale bar, 250 µm. *G*) *ADAM17* mRNA expression was down-regulated in DRG neuronal cultures from ADAM17^ex/ex^ mice (*n* = 3; Mann‐Whitney *U* test, *P* = 0.0027). *H*) Stacked histograms showing the proportions of different mechanosensitive currents in sensory neurons from ADAM17^+/+^ and ADAM17^ex/ex^ mice. Ablation of ADAM17 did not change the proportion of transient currents and SA currents in sensory neurons (χ^2^ test, *n* = 64–65, *P* = 0.95). *I*) Quantitative comparison of displacement threshold of mechano-gated currents in ADAM17^+/+^ and ADAM17^ex/ex^ sensory neurons. Displacement threshold was determined as mechanical stimulus that elicited a current ≥20 pA. Depletion of ADAM17 did not alter displacement threshold in DRG neurons (*P* = 0.86, unpaired Student’s *t* test, *n* = 43–44). *J*) Stimulus-response curve of mechanosensitive currents in ADAM17^+/+^ and ADAM17^ex/ex^ sensory neurons. Genetic depletion of ADAM17 did not affect mechanosensitive currents (*P* > 0.49, 2-way ANOVA with *post hoc* Bonferroni’s test, *n* = 52–54). Results are shown as means ± sem. *P < 0.05, **P < 0.01.

To determine whether the down-regulation of ADAM17 affects sensitivity to painful stimuli *in vivo*, we first investigated the behavioral responses of ADAM17^+/+^ and ADAM17^ex/ex^ mice to mechanical stimuli. ADAM17^ex/ex^ mice showed higher mechanical von Frey thresholds compared with ADAM17^+/+^ animals (**Fig. 3*A***; *n* = 8, 1-way repeated measurements ANOVA, *P* = 0.0156; Tukey’s *post hoc* test ADAM17^+/+^ *vs.* ADAM17^ex/ex^, *P* = 0.0477). We further assessed cutaneous morphology and innervation, because epithelial alterations, as previously reported for the mouse model, may alter the transfer of mechanical forces or temperature onto sensory nerve terminals and thus be causally involved in the generation of sensory deficits. ADAM17^ex/ex^ skin innervation pattern and epidermis thickness were similar to wild-type control mice (Fig. 3*B*): the density of free nerve endings was comparable for both mouse strains (ADAM17^+/+^: 3.08 ± 0.41 *vs.* ADAM17^ex/ex^: 2.59 ± 0.16 nerve ending per 1000 µm^2^; *n* = 4; *P* = 0.306, Student’s *t* test) and associated with normal epidermis thickness (35.10 ± 3.16 *vs.* 37.12 ± 2.54 µm; *n* = 4; *P* = 0.637, Student’s *t* test). These results suggest that the mechanical hyposensitivity in mice with low levels of ADAM17 was not associated with and unlikely to be caused by alterations of the overall skin structure or of the innervation pattern of cutaneous primary afferent nociceptive fibers.

In order to assess functional deficits of nociceptive afferents, C-fibers derived from the sural nerve of ADAM17^+/+^ and ADAM17^ex/ex^ mice were characterized in the skin nerve *ex vivo* preparation. We stimulated the receptive fields of the nerve fibers supplying the lateral outer side of the hind paw skin with voltage pulses, mechanical forces (M), heat (H), and cold (C) stimuli. C-fibers (C_) were identified by a conduction velocity smaller than 1 m/s and were similar in both mouse strains (Fig. 3*C*). Fibers were considered spontaneously active when 4 or more APs over 1 min were generated before applying any stimuli to the receptive field. C-fibers showing spontaneous activity were polymodal heat-sensitive fibers in nearly all cases. The percentage of spontaneously active fibers was similarly low in both genotypes (22 *vs.* 29%, *P* = 0.4296, Fisher’s exact test). In total, 51 C-fibers from 19 ADAM17^+/+^ and 88 fibers from 23 ADAM17^ex/ex^ mice were categorized as C_M, C_MH, C_MC, and C_MHC. The comparison of the 2 genotypes, with regard to the occurrence of different C-fiber types, yielded no significant difference (χ^2^ = 0.812; χ^2^ test, *P* = 0.8465, Fig. 3*D*). The mechanical threshold of mechanosensitive C-fibers was similar in both genotypes (ADAM17^+/+^: mean 19.65 ± 2.02 mN, median 16.00 mN, *n* = 51; ADAM17^ex/ex^: mean 22.98 ± 2.91 mN, median 13.70 mN, *n* = 88, *P* = 0.3814, Mann-Whitney *U* test), and this was also reflected by the frequency distribution of mechanical thresholds (Fig. 3*E*; *P* = 0.3034, χ^2^ = 4.846, χ^2^ test).

To determine whether the lack of ADAM17 influences mechanotransduction in sensory neurons, we recorded mechanosensitive currents using the whole-cell voltage-clamp configuration of the patch-clamp technique from cultured DRG neurons of ADAM17^+/+^ mice, which exhibit ADAM17 immunoreactivity in culture (Fig. 3*F*), and ADAM17^ex/ex^ mice, in which the enzyme is significantly down-regulated (Fig. 3*G*). Mechanosensitive currents evoked by indentation of the cell body with a blunt glass probe can be classified as rapidly adapting (RA, τ1 <5 ms), intermediate-adapting (IA, 5 ms < τ1 < 50 ms) and slowly adapting (SA, τ1 >50 ms or no adaptation during a 500 ms stimulus) based on the inactivation time constant τ1 (34, 38). Because increasing the stimulus magnitude sometimes induced RA currents to become IA currents (39), we combined RA and IA into 1 group and defined them as transient currents. Approximately 83% (54 out of 65 cells) of DRG neurons had either transient currents or SA currents in ADAM17^+/+^ sensory neurons, and this was similar in neurons from ADAM17^ex/ex^ mice (81%; 52 out of 64 cells; Fig. 3*H*). Moreover, both ADAM17^+/+^ and ADAM17^ex/ex^ sensory neurons had similar displacement thresholds (Fig. 3*I*; ADAM17^+/+^: 4.0 ± 0.25 µm, *n* = 44; ADAM17^ex/ex^: 4.07 ± 0.25 µm, *n* = 43). We next plotted the amplitude of mechanically gated currents (transient currents and SA currents) against the increasing displacement for both ADAM17^+/+^ and ADAM17^ex/ex^ groups. Analysis of displacement-response curves revealed that ablation of ADAM17 did not alter mechanosensitive currents (Fig. 3*J*; 2-way ANOVA, *P* > 0.49).

Collectively, these results indicated that the genetic reduction of ADAM17 protein levels caused hyposensitivity to mechanical stimuli but, however, did not change the mechanosensitivity of primary afferent nociceptors.

### Down-regulated ADAM17 is associated with thermal hyposensitivity

**Figure 4:**
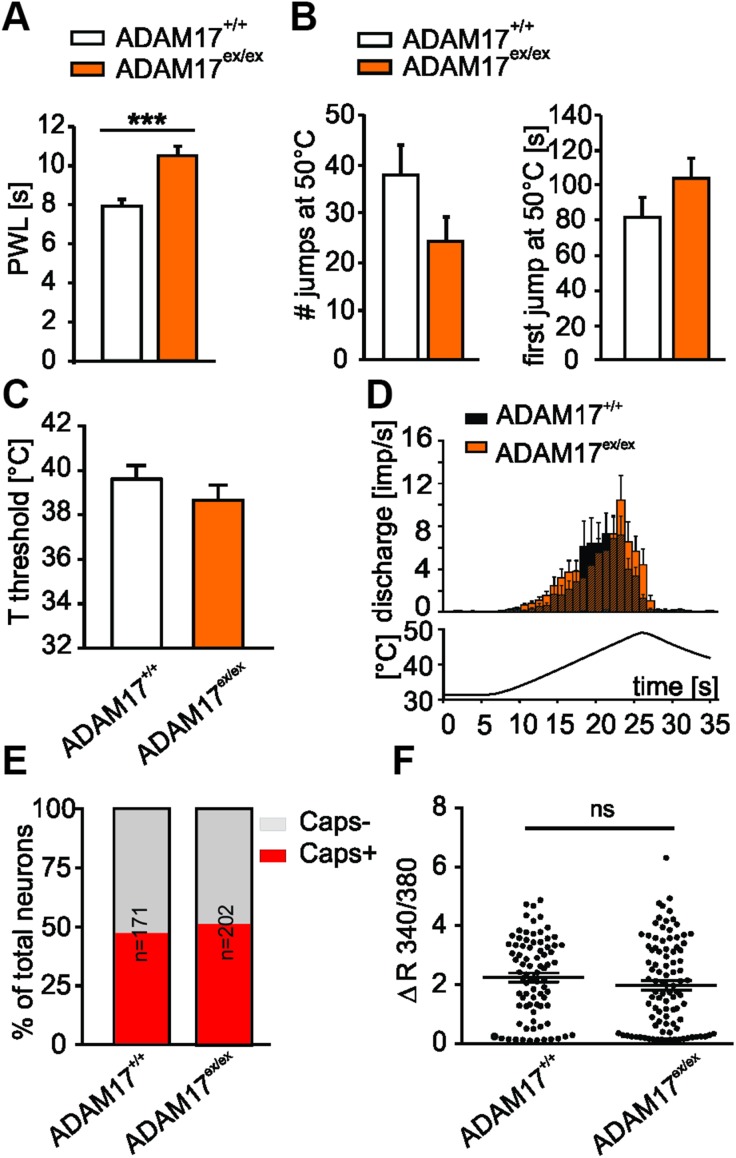
ADAM17^ex/ex^ mice show reduced sensitivity to noxious heat stimuli *in vivo*. *A*) Hargreaves test conducted on ADAM17^ex/ex^ and control mice showed that hypomorphic mice were hyposensitive to heat stimuli compared with controls (10.51 ± 0.49 *vs.* 7.92 ± 0.36 s; *n* = 12; *P* < 0.001, Kruskall-Wallis test, followed by Mann-Whitney *U* *post hoc* test). PWL, paw withdrawal latency. *B*) Both genotypes showed similar behavior in heat responses within 3 min recordings on the hot plate test (ADAM17^ex/ex^ *vs.* ADAM17^+/+^ jumps *n* = 24.23 ± 5.04 *vs.* 37.86 ± 6.27, *P* = 0.106; first jump 103.44 ± 12.12 *vs.* 81.28 ± 11.[^35^S], *P* = 0.085; ADAM17^+/+^ *n* = 14, ADAM17^ex/ex^ *n* = 13; Mann-Whitney *U* test). *C*) Temperature threshold was similar in both genotypes (39.6 ± 0.6°C for ADAM17^+/+^ and 38.7 ± 0.7°C for ADAM17^ex/ex^, *P* = 0.3149, unpaired Student’s *t* test). *D*) Heat-sensitive C-fibers from ADAM17^ex/ex^ mice responded to a standard ramp heat stimulus with a similar rate of discharge compared with those from control mice (3.34 ± 0.51 imp/s for ADAM17^ex/ex^, *n* = 40 *vs.* 2.89 ± 0.82 imp/s for control mice, *n* = 30), *P* = 0.211, Mann-Whitney *U* test). *E*) Calcium imaging was used to record capsaicin-evoked Ca^2+^ transient in ADAM17^+/+^ and ADAM17^ex/ex^ DRG neurons. Stacked histograms show that there was no difference in the percentage of capsaicin-responding (Caps^+^) neurons *vs*. non–capsaicin-responding (Caps^−^) neurons between genotypes (Fisher’s exact test, *n* = 171–202, *P* > 0.9999). *F*) Dot-plot graph showing no significant difference in capsaicin-evoked Ca^2+^ increase in magnitude between control and ADAM17^ex/ex^ neurons (Mann-Whitney *U* test, *n* = 81–95, *P* = 0.2228). Each dot represents a single capsaicin-responding cell. Results are shown as means ± sem. Ns, not significant. ***P < 0.001.

Moreover, ADAM17^ex/ex^ mice were found to be hyposensitive to heat stimuli compared with controls. Responsiveness to thermal stimuli was reduced in ADAM17^ex/ex^ mice, which showed significantly increased withdrawal latencies to radiant heat stimulation (**Fig. 4*A***; 10.51 ± 0.49 *vs.* 7.92 ± 0.36 s; *n* = 12; *P* < 0.001, Kruskall-Wallis test, followed by Mann-Whitney *U* *post hoc* test). However, both genotypes showed similar behavior in the hot plate test (Fig. 4*B*), and the temperature thresholds of heat-sensitive C-fibers derived from ADAM17^+/+^ (39.6 ± 0.6°C) and ADAM17^ex/ex^ mice (38.7 ± 0.7°C) were similar (Fig. 4*C*; *P* = 0.3149, unpaired Student’s *t* test). Responses to a standard ramp-shaped heat stimulus at the receptive field was also comparable in both genotypes (Fig. 4*D*; 3.34 ± 0.51 discharge frequency in impulses/second (imp/sec) for ADAM17^ex/ex^, *n* = 40 *vs.* 2.89 ± 0.82 imp/s for control mice, *n* = 30, *P* = 0.211, Mann-Whitney *U* test). The characterization and comparison of C-fibers derived from ADAM17^+/+^ and ADAM17^ex/ex^ mice showed no differences regarding heat temperature thresholds, their distribution, or firing rates indicative of a change in heat transduction mechanisms. Because the TRPV1 signal transducer ion channel is considered to be an important sensor for noxious heat detection (40), we used the Fura-2–based ratiometric calcium imaging technique to determine the effect of ADAM17 on the neuronal response to TRPV1 agonists. There was no difference in TRPV1 responses between genotypes; the proportion of neurons that responded to the TRPV1 agonist capsaicin (250 nM 10 s) was comparable between genotypes (Fig. 4*E*; Fisher’s exact test, ADAM17^+/+^: 81 of 171 cells; ADAM17^ex/ex^: 95 of 202 cells, *P* > 0.9999), and the magnitude of capsaicin-induced Ca^2+^ transients was also similar (Fig. 4*F*; Mann-Whiney *U* test, ADAM17^+/+^: Δ*F*_340/380_ = 2.25 ± 0.15; ADAM17^ex/ex^: Δ*F*_340/380_ = 1.99 ± 0.15, *n* = 81–95, *P* = 0.2228), suggesting that depletion of ADAM17 did not affect TRPV1-mediated capsaicin response in sensory neurons.

**Figure 5:**
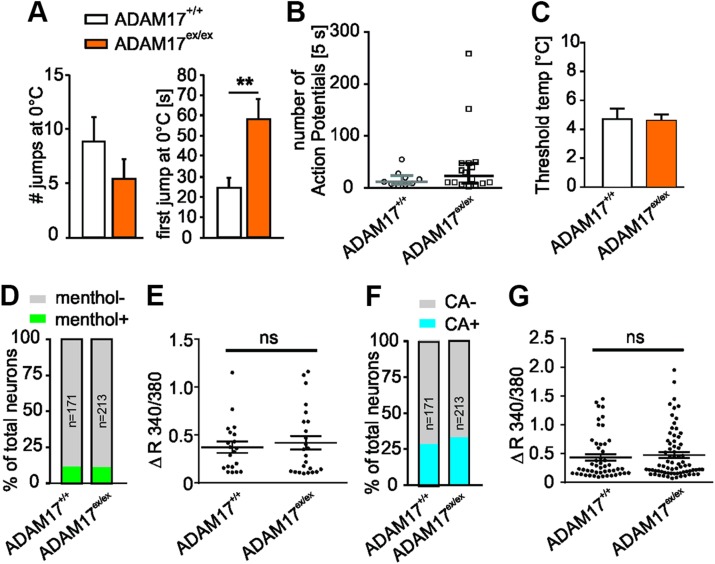
ADAM17^ex/ex^ mice show reduced sensitivity to noxious cold stimuli *in vivo*. *A*) Responses to cold within 3 min recordings were reduced in ADAM17^ex/ex^ mice, which showed significantly higher time span to execute the first jump after 0°C stimuli (58.22 ± 9.91 *vs.* 24.32 ± 5.08 s; ADAM17^+/+^ *n* = 14, ADAM17^ex/ex^ *n* = 13; *P* = 0.006, Mann-Whitney *U* test). *B*) Depletion of ADAM17 did not affect the rate of discharge of cold-sensitive C-fiber afferents to cold stimuli (median: 12.00 *vs.* 23.50 APs (first 5 s); ADAM17^+/+^ *n* = 9, ADAM17^ex/ex^ *n* = 16; *P* = 0.3074, Mann-Whitney *U* test). *C*) Threshold cold temperatures of cold-sensitive C-fiber afferents was comparable between the 2 genotypes (4.76 ± 0.67 *vs.* 4.64 ± 0.40°C; ADAM17^+/+^ *n* = 9, ADAM17^ex/ex^ *n* = 16; *P* = 0.8730, unpaired Student’s *t* test). *D*, *F*) Stacked histograms showing that the proportion of menthol-responding (menthol^+^) and non–menthol-responding (menthol^−^) neurons (*D*) and CA-responding (CA^+^) and non–CA-responding (CA^−^) neurons (*F*) was comparable between control and ADAM17^ex/ex^ neurons (Fisher’s exact test, *n* = 171–213, *P* > 0.05). *E*, *G*) The dot-plot graph showing no significant difference in both menthol-evoked Ca^2+^ increase magnitude [(*E*), Mann-Whitney *U* test, *n* = 20–24, *P* = 0.8795)] and CA-evoked Ca^2+^ increase magnitude [(*G*), Mann-Whitney *U* test, *n* = 49–71, *P* = 0.5846)] between genotypes. Results are shown as means ± sem; ns, not significant. **P < 0.01.

More evidence for a general reduction in somatosensitivity emerged from the finding that mice with a down-regulation of ADAM17 showed significantly higher latencies of the first jump in the cold plate test (58.22 ± 9.91 *vs.* 24.32 ± 5.08 s; ADAM17^+/+^ *n* = 14, ADAM17^ex/ex^ *n* = 13; *P* = 0.006, Mann-Whitney *U* test, **Fig. 5*A***). However, recordings from cold-sensitive C-fiber afferents did not show differences in cold responses (Fig. 5*B*) or threshold cold temperatures between the 2 genotypes (Fig. 5*C*). We next determined whether depletion of ADAM17 affects the thermal response at the transduction level. Transient receptor potential cation channel, subfamily M, member 8 (TRPM8) and subfamily A, member 1 (TRPA1) have been identified to act as cold sensors in the somatosensory system (41, 42). In ADAM17^+/+^ cultures, around 11.7% (20 of 171) of neurons responded to the stimuli of TRPM8 agonist menthol (1 mM, 60 s) with a Ca^2+^ transient, and 28.6% (49 of 171) of cells responded to TRPA1 agonist CA (200 µM, 90 s). Similar proportions of neurons that displayed a response to 1 mM menthol (Fig. 5*D*; ADAM17^ex/ex^: 11.3%, 24 of 213) and 200 µM CA (Fig. 5*F*; ADAM17^ex/ex^: 33.3%, 71/213) were observed in ADAM17^ex/ex^ cultures. In addition, depletion of ADAM17 did not alter the peak amplitude of both menthol- and CA-induced Ca^2+^ transients (Fig. 5*E, G*; Mann-Whiney *U* test; menthol-induced Δ*F*_340/380_, 0.37 ± 0.06 *vs.* 0.42 ± 0.07, *n* = 20–24, *P* = 0.8795; CA-induced Δ*F*_340/380_, 0.44 ± 0.06 *vs.* 0.48 ± 0.05, *n* = 49–71, *P* = 0.5846). These data suggested that ADAM17 did not regulate the activity of TRPM8 and TRPA1 in sensory neurons.

### Down-regulated ADAM17 affects specific biophysical properties

**Figure 6:**
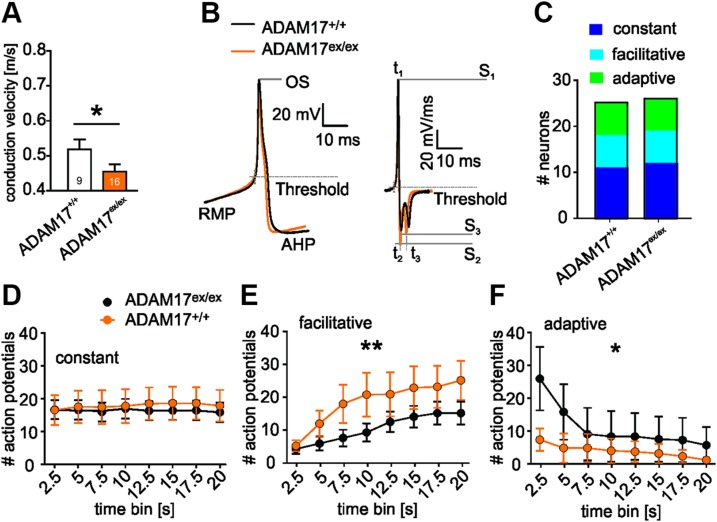
Down-regulated ADAM17 affects neuron biophysical properties. *A*) The average conduction velocity of cold-sensitive C-fibers in ADAM17^ex/ex^ skin-nerve recordings was reduced compared with that of ADAM17^+/+^ cold-responsive C-fibers (ADAM17^+/+^ *n* = 12 and ADAM17^ex/ex^ *n* = 20, *P* = 0.047, Student’s *t* test). *B*) Representative AP recording from an ADAM17^+/+^ and ADAM17^ex/ex^ DRG nociceptive neuron and its first derivative used for AP characterization; the shape and AP kinetics were comparable between the 2 genotypes. AP parameters, *e.g.*, RMP, AHP, threshold, AP speed of depolarization (*S*_1_) and repolarization (*S*_2_, *S*_3_), and the corresponding time points (*t*_1_, *t*_2_, *t*_3_) were analyzed OS, overshoot. *C*) Distribution of ADAM17^+/+^ and ADAM17^ex/ex^ sensory neurons with constant, adaptive, or facilitative firing patterns did not differ between both genotypes (χ^2^ test, *P* = 0.9981). *D*) Number of generated APs (averaged over 2.5 s time bins) of the constant firing ADAM17^ex/ex^ sensory neurons was unaltered (2-way ANOVA, genotype factor *P* = 0.8061, ADAM17^+/+^ *n* = 11 and ADAM17^ex/ex^ *n* = 12). *E*, *F*) Whereas ADAM17^ex/ex^ sensory neurons showed an increased facilitative firing frequency in comparison with ADAM17^+/+^ sensory neurons (*E*; 2-way ANOVA, genotype factor *P* < 0.01, ADAM17^+/+^ *n* = 7 and ADAM17^ex/ex^ *n* = 7), the adaptive firing frequency of ADAM17^ex/ex^ sensory neurons (*F*) was significantly lower than that of their ADAM17^+/+^ counterparts (2-way ANOVA, genotype factor *P* < 0.05, ADAM17^+/+^ *n* = 7 and ADAM17^ex/ex^ *n* = 7). Results are shown as means ± sem. *P < 0.05, **P < 0.01.

Given that ADAM17^ex/ex^ mice presented hyposensitivity to mechanical as well as thermal stimuli but no corresponding difference in sensory neuron responses or activation thresholds, we hypothesized that the phenotype could be due to a general alteration in electrical properties, such as excitability. Although conduction velocities of heat-responsive unmyelinated primary afferents were comparable for ADAM17^ex/ex^ and control mice, cold-sensitive fibers (C_MC and C_MHC) conducted with a significantly slower velocity in ADAM17^ex/ex^ mice (ADAM17^+/+^: 0.50 ± 0.024 *vs.* ADAM17^ex/ex^: 0.44 ± 0.020 m/s, Student’s *t* test, *P* = 0.0473, **Fig. 6*A***), independent of whether these fibers showed spontaneous activity or not. As this may be indicative of a possibly reduced excitability of sensory neurons in the ADAM17 depleted mice, we hypothesized that the hyposensitivity to mechanical and thermal stimulation could be due to a general decrease in biophysical parameters setting the overall excitability of sensory neurons. Therefore, we investigated the passive and active membrane properties of ADAM17^ex/ex^ DRG nociceptive neurons, such as AP kinetics, and the different parameters obtained from the first derivative of the AP of control and ADAM17-depleted mice (Fig. 6*B* and **Table 1**). The threshold to evoke an AP was significantly elevated in ADAM17^ex/ex^ sensory neurons (ADAM17^+/+^ −29.044 ± 0.949 mV *vs.* ADAM17^ex/ex^ −32.651 ± 0.662 mV, *P* = 0.002, 2-sample Student’s *t* test), whereas none of the other obtained parameters showed a significant difference between genotypes. In addition, we investigated AP firing of cultured sensory neurons evoked by a 20 s depolarizing current injection, which generates 3 different types of firing patterns in sensory neurons (32). The occurrence of constant, adaptive, and facilitative firing patterns, as well as the constant firing response, was similar in both genotypes (Fig. 6*C*, *D*). However, within the constant firing population of ADAM17^ex/ex^ sensory neurons, we observed a significant hyperpolarizing shift of the RMP and AP threshold (Table 1). In addition, genotype-specific differences were observed in both adaptive and facilitative firing sensory neurons (Fig. 6*E*, *F*). A slightly more pronounced AHP was obtained in ADAM17^ex/ex^, which, however, was not statistically significant (ADAM17^ex/ex^ −68.440 ± 0.685 *vs.* ADAM17^+/+^ −66.179 ± 0.943, *P* = 0.054, 2-sample Student’s *t* test). Taken together, these alterations of biophysical attributes are in line with a reduced excitability of sensory neurons in ADAM17^ex/ex^ mice, which exhibit pronounced sensory deficits, *in vivo*.

**TABLE 1:** AP characteristics of nociceptive neurons based on their firing pattern

	General			Constant			Adaptive			Facilitative		
Parameter	WT (*n* = 25)	ADAM17^ex/ex^ (*n* = 29)	*P*	WT (*n* = 11)	ADAM17^ex/ex^ (*n* = 12)	*P*	WT (*n* = 7)	ADAM17^ex/ex^ (*n* = 7)	*P*	WT (*n* = 7)	ADAM17^ex/ex^ (*n* = 7)	*P*
IR (GΩ)	1.427 ± 0.104	1.288 ± 0.093	0.325	1.472 ± 0.142	1.344 ± 0.118	0.496	1.198 ± 0.166	1.137 ± 0.197	0.817	1.587 ± 0.246	1.343 ± 0.201	0.458
Rheobase (pA)	33.000 ± 7.466	28.103 ± 5.195	0.585	10.909 ± 1.131	23.75 ± 9.068	0.193	62.142 ± 21.48	35 ± 12.34	0.295	38.571 ± 7.536	33.571 ± 9.110	0.680
RMP (mV)	−50.487 ± 1.343	−51.744 ± 0.903	0.430	−45.838 ± 1.519	−50.340 ± 1.153	0.027*	−52.945 ± 2.473	−53.011 ± 1.964	0.984	−55.334 ± 1.741	−53.803 ± 1.874	0.561
OS (mV)	67.642 ± 0.818	67.254 ± 0.863	0.748	67.988 ± 1.295	68.981 ± 0.734	0.503	64.859 ± 1.497	64.246 ± 2.544	0.839	69.881 ± 0.873	66.872 ± 1.914	0.178
AHP (mV)	−66.179 ± 0.943	−68.440 ± 0.685	0.054	−68.457 ± 1.049	−70.724 ± 0.593	0.068	−60.650 ± 1.192	−65.653 ± 1.993	0.052	−68.128 ± 1.149	−67.241 ± 0.907	0.556
τAHP (ms)	108.829 ± 17.841	98.878 ± 10.032	0.614	79.314 ± 7.9002	98.510 ± 17.689	0.348	76.995 ± 4.8481	72.500 ± 7.9993	0.639	200.080 ± 58.087	119.900 ± 22.729	0.200
AP threshold (mV)	−29.044 ± 0.949	−32.651 ± 0.662	0.002*	−28.135 ± 1.853	−33.376 ± 1.049	0.020*	−28.374 ± 1.268	−30.952 ± 1.437	0.203	−31.143 ± 1.114	−32.878 ± 1.233	0.317
*S*_1_ (mV/ms)	186.431 ± 13.385	197.996 ± 10.033	0.486	196.236 ± 21.829	220.005 ± 13.513	0.356	150.338 ± 24.266	177.548 ± 28.751	0.483	207.117 ± 20.227	185.476 ± 10.014	0.357
*S*_2_ (mV/ms)	−37.272 ± 2.912	−34.867 ± 2.698	0.547	−37.388 ± 5.436	−34.303 ± 2.614	0.605	−38.654 ± 5.633	−28.577 ± 3.272	0.148	−35.706 ± 3.366	−45.023 ± 8.982	0.351
*S*_3_ (mV/ms)	−30.622 ± 2.103	−33.694 ± 1.979	0.293	−34.761 ± 3.107	−37.048 ± 2.141	0.545	−22.449 ± 2.550	−28.887 ± 5.215	0.289	−32.290 ± 3.919	−32.475 ± 4.398	0.975
*t*_1_–*t*_2_ (ms)	1.572 ± 0.101	1.542 ± 0.090	0.821	1.688 ± 0.168	1.495 ± 0.110	0.343	1.413 ± 0.188	1.847 ± 0.157	0.102	1.551 ± 0.168	1.287 ± 0.253	0.403
*t*_1_–*t*_3_ (ms)	5.152 ± 0.289	5.008 ± 0.210	0.684	5.105 ± 0.497	4.859 ± 0.223	0.647	4.838 ± 0.372	5.206 ± 0.322	0.469	5.54 ± 0.605	4.961 ± 0.760	0.563
*t*_2_–*t*_3_ (ms)	3.579 ± 0.223	3.466 ± 0.149	0.669	3.417 ± 0.360	3.363 ± 0.160	0.889	3.425 ± 0.239	3.358 ± 0.193	0.834	3.988 ± 0.527	3.673 ± 0.548	0.686
RMP-AP threshold (mV)	−21.442 ± 1.180	−19.092 ± 0.894	0.113	−17.702 ± 1.328	−16.963 ± 1.241	0.688	−24.571 ± 2.281	−22.058 ± 1.960	0.420	−24.190 ± 1.873	20.925 ± 1.159	0.164

Values are means ± sem, with sample size in parentheses. *P* values were determined by 2-sample Student’s *t* tests. τAHP, AHP recovery time constant; IR, input resistance; OS, overshoot; *S*_1_, depolarization velocity; *S*_2_, first repolarization peak velocity; *S*_3_, second repolarization peak velocity; *t*_1_–*t*_2_, time interval between *S*_1_ and *S*_2_; *t*_1_–*t*_3_, time interval between *S*_1_ and *S*_3_; *t*_2_–*t*_3_, time interval between *S*_2_ and *S*_3_; WT, wild type. *P < 0.05.

## DISCUSSION

Relevant pain mediators and their receptors, such as TNF-α, IL-6 receptor, or fractalkine, are shed by ADAM17, which is a member of the adamalysin protein family that modulates cell surface proteins in important events from proliferation to migration. In the present study, we have shown for the first time that ADAM17 is critically involved in setting the sensitivity to noxious mechanical, cold, and heat stimuli *in vivo*; however, the underlying mechanisms, apparently, do not rely on alterations of primary nociceptive afferent morphology or transduction of sensory stimuli but are associated with specific changes affecting nociceptor excitability and the loss of a small subpopulation of nonpeptidergic IB4-positive nociceptors.

In the nervous system, ADAM17 is expressed in neurons, astrocytes, immune cells, and endothelium (43, 44). Although a global deletion of Adam17 is lethal, hypomorphic ADAM17^ex/ex^ mice with drastically reduced shedding of ADAM17 substrates exhibit compromised eye and heart development and skin defects without, however, any changes in the brain, body segmentation, and vascular development (2, 3). Our data, for the first time, to our knowledge, reported *ADAM17* mRNA and protein expression in DRG neurons and in the spinal cord, indicating a possible involvement of ADAM17 in pain processes. In our present study, the gross skin architecture, the cutaneous innervation pattern of nociceptors, and DRG and spinal cord morphology were unaltered, pointing to normal development of large parts of the pain pathway in mice with loss of ADAM17 activity. This is surprising, because ADAM17 through juxtamembrane proteolysis of neurotrophin and reticulon receptors was reported to stimulate axonal growth (45, 46). Despite the normal DRG morphology, the small but significant loss of IB4-positive neurons may suggest that at least a particular subpopulation of small-diameter sensory neurons requires growth regulators that are specifically shed by ADAM17. Even if the decrease in the proportion of nonpeptidergic primary afferent neurons may not appear to be sufficiently large to fully explain the difference in behavior, our finding is in line with previous studies reporting that ablation of IB4-positive neurons transiently reduces both mechanical and heat sensitivity (47, 48).

Although ADAM17 has so far not been explored in the pain pathway, several targets of the enzyme, such as TNF-α, IL-6, or the IL-6 signal transducer gp130, are generally accepted as critically important regulators for the induction and maintenance of nociceptor sensitization to mechanical and heat stimuli in mouse models of pathologic pain (12–16). In particular, mice lacking gp130 are protected from the pain hypersensitivity associated with tumor, nerve injury, or inflammation, and they exhibit significantly lower mechanical thresholds compared with control mice (13, 16). Additional evidence for a possible involvement of ADAM17 and IL-6 *trans*-signaling in the pain pathway comes from transgenic mice which express the IL-6 tran-signaling inhibitor sgp130Fc under the transcriptional control of the phosphoenolpyruvate carboxykinase promoter. These animals spend longer time on the hot plate (49). Sun *et al*. (18) showed that the Notch intracellular domain, which is cleaved at the juxtamembrane S1 site by ADAM10 and possibly by ADAM17, is up-regulated in the sciatic nerve and spinal cord in a neuropathic pain model of nerve injury; moreover, inhibition of Notch signaling pathway increases paw withdrawal thermal latency and mechanical threshold and reverses hyperalgesia after nerve injury.

In line with these reports, mechanical and thermal sensitivity was considerably reduced in ADAM17^ex/ex^ mice compared with control animals. Nonpeptidergic IB4-positive neurons express the mechanosensitive transducer ion channel TRPA1, and the mechanical hyposensitivity of nociceptor-specific gp130 deficient mice is associated with a reduced expression of TRPA1 (20). IA and SA mechanosensitive ionic currents are reduced in DRG neurons with a *Trpa1* null mutation (39, 50). Surprisingly, DRG cultures from the ADAM17 hypomorphic mice did not show changes in mechanically activated currents or TRPV1-, TRPM8-, or TRPA1-mediated calcium transients, neither mechanical nor thermal response parameters of cutaneous C-fibers. Changes in those parameters would in fact correspond to the mechanical or thermal hyposensitivity observed *in vivo*. It is important, however, to notice that the *in vitro* stimulus, which corresponds to cell bodies’ poking, might differ from the *in vivo* stimulus, which stimulates the axons innervating the skin. Nevertheless, our findings suggest that the regulation of TRPA1 and other transducer ion channels in nociceptive neurons likely occurs independently of ADAM17. Rather, the *in vivo* hyponociceptive phenotype resulting from ADAM17 down-regulation may depend on other cellular and molecular mechanisms requiring the shedding of ADAM17 targets.

The ADAM17 target IL-6 receptor, *via* its signal transducer gp130, acts as a brake for the expression of potassium channels and is an important regulator hub for nociceptor excitability (31). Deletion of gp130 alters excitability parameters that are linked to changes in potassium conductance; in particular, in sensory neurons lacking gp130, A-type K^+^ currents and potassium voltage-gated channel subfamily A member 4 (Kcna4) expression increase, resulting in a hyperpolarized RMP. In line with these findings, ADAM17 reduces the expression of voltage-dependent potassium channels (9). In the present study, several biophysical parameters, including the conduction velocity of cold-sensitive C-fibers, the RMP, AHP, and firing patterns, were altered in distinct nociceptor modalities in ADAM17^ex/ex^ mice. A plausible explanation for these changes lies in the lack of ADAM17 and important regulators shed by the enzyme, which in turn could release the brake for the expression of potassium channels. Increased activity of potassium currents would hyperpolarize the RMP and reduce the probability of neuron excitation, leading to reduced responsiveness to painful stimuli *in vivo.*

Changes in spinal cord morphology may be a signature for differences in pain transmission. However, the overall architecture of the spinal cord and the distribution of nociceptive fibers in the spinal dorsal horn were unaltered in ADAM17^ex/ex^ mice. In the CNS, immune-like glial cells, such as microglia, support normal sensory transmission and nociception, whereas in pain pathologic conditions, immune responses are triggered and microglia are activated (51, 52). We observed that mice lacking ADAM17 showed hyposensitivity to painful stimuli; however, the number of activated microglia and astroglia were comparable to those in wild-type mice, suggesting that the phenotype observed *in vivo* cannot be sufficiently explained by a regulatory function on spinal dorsal horn morphology or glia. ADAM proteins, in particular ADAM10, are detected also in the cerebral cortex, hippocampus, and ventral hypothalamus (53), where they and some of their targets are involved in CNS development (37), learning and memory (54, 55), and depression (56). Thus, the control of pain sensitivity involving ADAM proteins might be linked to changes in higher anatomic structures in the CNS rather in the peripheral nervous system. In particular for IL-6 *trans*-signaling, differential and partially opposing effects are known in the CNS *vs.* the peripheral nervous system: ADAM17 cleaves proteins implicated in learning and memory, like the neuronal pentraxin, which binds α-amino-3-hydroxy-5-methyl-4-isoxazolepropionic acid (AMPA)-type glutamate receptors necessary for long term depression in hippocampal and cerebellar synapses (55) as well as the cell adhesion molecule 1 (Cadm1) that participates in synaptic connection formation and plasticity (57). The frequency of excitatory postsynaptic currents in cortical areas is higher in the absence of IL-6 *trans*-signaling (58), and IL-6 mRNA is down-regulated in the absence of ADAM17 (6).

An increasing number of reports support the concept that the peripheral immune system acts on the brain to alter an individual’s response to stress, ultimately contributing to vulnerability to mood disorders (59). In a model of major depressive disorder, TNF-α expression is increased, and an antidepressant treatment decreases the expression of ADAM17 and its targets CXCL2 and IL-6. These changes shift the balance between synaptic inhibition and excitation in favor of the latter in the prefrontal cortex, and they may thus increase descending pain inhibitory pathways (56, 60, 61). Thus, an increased activity in pain-controlling brain areas with increased descending inhibition of spinal dorsal horn circuits could possibly contribute to the reduced pain behavior in the ADAM17^ex/ex^ mice in the present study.

Recent literature, together with our present findings, suggests the activation of complex mechanisms along the pain pathway, involving ADAM17 and the shedding of important regulators of peripheral neuron excitability, as well as of synaptic processes in the CNS. For the first time, our study supports the idea that ADAM17 acts as an important regulator orchestrating the sensitivity to noxious stimuli and could potentially serve as an important new target for interventions, aiming at a general reduction of signal processing in the pain pathway.

## Abbreviations

ADAM: A disintegrin and metalloproteinase
ADAM17^+/+^: ADAM17 wild type mice
ADAM17^ex/ex^: hypomorphic ADAM17 mice
AHP: afterhyperpolarization
AP: action potential
C-fiber: unmyelinated nociceptor
CA: cinnamaldehyde
CGRP: calcitonin-related peptide
DRG: dorsal root ganglia
EGF-R: endothelial growth factor receptor
gp130: glycoprotein 130
IA: intermediate adapting
*I*_AP_: minimal current injection to evoke a single AP
IB4: isolectin β-4
imp/s: impulses/second
NF200: neurofilament 200
RA: rapidly adapting
RMP: resting membrane potential
SA: slowly adapting
TACE: TNF-α-converting enzyme
TRPA1: transient receptor potential cation channel, subfamily A, member 1
TRPM8: transient receptor potential cation channel, subfamily M, member 8
TRPV1: transient receptor potential cation channel, subfamily V, member 1
